# Can Ikigai Predict Anxiety, Depression, and Well-being?

**DOI:** 10.1007/s11469-022-00764-7

**Published:** 2022-03-01

**Authors:** Juliet Wilkes, Gulcan Garip, Yasuhiro Kotera, Dean Fido

**Affiliations:** Enterprise Centre, University of Derby Online Learning, University of Derby, Bridge Street, Derby, DE1 3LD UK

**Keywords:** Ikigai, Anxiety, Depression, Well-being, General population

## Abstract

The Japanese construct of ikigai reflects a sense of having ‘purpose in life’ or a ‘reason for living and has been associated with a variety of positive health outcomes. However, to date little research into ikigai exists within Western populations. This study explored the predictive power of ikigai for measures of well-being, depression, and anxiety in an adult Western population. Ninety-four participants (70% female) responded to an online survey. After accounting for the covariates of sex, age, employment status, and student status, multiple hierarchical regression indicated that ikigai positively predicted well-being and negatively predicted depression. While on its own, ikigai negatively predicted anxiety; this was not the case after accounting for the aforementioned covariates. The findings support the importance for investigating ikigai in the West and the need for further exploration of ikigai as a potential means of bringing about benefit in mental well-being.

Research addressing mental health and well-being is vital. Depression, characterised by persistent sadness, lack of interest, and/or poor concentration, is the leading cause of disability worldwide, and anxiety, typified by habitual unease and worry, is the main symptom of anxiety disorders which are the second most widespread worldwide mental health disorders (World Health Organisation, 2017). Not only can poor mental health have a negative impact on the individual and their loved ones but the global financial cost of poor mental health predicted to be 2.5 trillion dollars per year (The Lancet Global Health, 2020). Interestingly, lifetime rates of clinical depression and anxiety have been found to be up to ten times more in the West than in Asia (De Vaus et al., 2018). This could in part be due to a cultural difference, whereby people in the West have been found to be more willing to disclose mental health (Kotera et al., 2020). While it is important to consider this, it may not fully explain away such a reported difference. Therefore, delineating some of the potential underpinnings of such a disparity might be useful in developing a better understanding of and treatment pertaining to mental health.

Research into one’s meaning in life has proliferated in recent years (Martela & Steger, 2016). While much focus has been placed on coherence (‘the feeling that one’s experiences or life itself makes sense’; Heintzelman & King, 2014, p. 154) and purpose (‘a sense of core goals, direction in life, and enthusiasm regarding the future’; George & Park, 2013, p. 371), there exists a lesser studied, yet still important meaning in life significance. Significance encompasses the worthwhileness and value of one’s life (Martela & Steger, 2016). While this is associated with the Greek concept of *eudaimonia* (living well, successfully, and responsibly), research on eudaimonia concentrates on what *leads* to a life worth living, whereas significance is specifically concerned with the *experience* (Martela & Steger, 2016). This experiential concept is directly connected to the Japanese construct of ‘ikigai’ (生き甲斐) reflecting a sense of having ‘purpose in life’ or a ‘reason for living’ (Mathews, 1996; Mori et al., 2017; Park, 2015).

Research from Japan indicated that the presence of ikigai is associated with a variety of health benefits, including better self-reported physical health (Murata et al., 2006), lower rates of mortality (Sone et al., 2008; Tanno et al., 2009), reduced functional disability (Mori et al., 2017), and decreased psychological burden in caregivers (Okamoto & Harasawa, 2009). Although the precise mechanism by which ikigai is related to mental health is yet unknown, these findings do start to indicate that ikigai may be a potential mechanism by which mental health status may be predicted. However, this literature is limited due to its use of varying definitions of ikigai and ill-defined descriptions of ikigai measures (Hasegawa et al., 2015). Further, research in this field has often measured ikigai singularly by asking an individual to indicate if they feel they have ikigai or not (Murata et al., 2006; Okamoto & Harasawa, 2009; Tanno et al., 2009), which fails to capture the different types or degrees of ikigai that may be present in individuals. Overcoming this, Imai et al. (2012) developed and validated the Ikigai-9, which measures ikigai over three key dimensions, namely (i) optimistic and positive emotions toward life, (ii) active and positive attitudes towards one’s future, and (iii) acknowledgment of the meaning of one’s existence. More recently, Fido et al. (2019) have translated this into English; finding that a single factor solution better represented the conceptualisation of ikigai in a UK population. Although other measures of ikigai do exist, they lack the breadth displayed by Ikigai-9 (Kondo & Kamada, 2003) or fail to capture the most commonly accepted features of ikigai by focusing heavily, for example, on enjoyment from leisure pursuits (Kono et al., 2019). Although little empirical investigation into ikigai has been conducted outside of Japan, Fido et al. (2019) identified significant relationships between ikigai and both well-being and depression (but not anxiety or stress) in UK-based participants after controlling for sex and age. Though an important first step in understanding the manifestation of ikigai within the West, a major critique of this study is that the Depression Anxiety Stress Scale (DASS-21; Lovibond & Lovibond, 1995) used lacked the scope and sensitivity to sufficiently detect any differences in stress or anxiety present in the sample if indeed they exist. While previous studies have used confirmatory factor analysis to address the validity of DASS-21, Lee (2019) argues the majority display limitations by yielding poor factor loading values and further by failing to address discriminant validity (i.e., if the variables can be considered separate from each other) and nomological validity (i.e., if the DASS-21 matched the theoretical assertions of the literature). Furthermore, a recent biological study exploring depression positioned stress as one contributing factor to depression (Kobayashi et al., 2020). Fido et al. (2019) indicate that further research looking at the predictive value of ikigai for mental health and well-being in a Western population is needed, and as such, said research may benefit from using alternative scales to the DASS-21 to measure anxiety, depression, and stress.

The aim of this study is to ascertain if ikigai is correlated with measures of mental health in a Western population. We base this research on the seminal work of Fido and colleagues (2019). However, to overcome the limitation discussed above, we use a more comprehensive measure of well-being. As the factors, age, gender, employment status, and student status, have been found to be associated with depression, anxiety, and well-being levels (Boyd et al., 2015; Gray et al., 2019; Ibrahim et al., 2013; Jefferis et al., 2011; McManus et al., 2016; Neves & Hillman, 2017; Paul & Moser, 2009; Steptoe et al., 2015), these demographic variables are accounted for within the study to ensure any observed variance is explained by ikigai above and beyond these factors. Accounting for these variables also allows the study to determine if there are any differences in levels of ikigai based on gender and for the first time in a Western sample, employment, and student status. The research questions are as follows: is ikigai correlated with measures of depression; is ikigai correlated with measures of well-being; and, is ikigai correlated with measures of anxiety? Based on the research discussed within, it is hypothesised that participants who report a high sense of ikigai will have lower depression scores and higher well-being scores compared to participants who have a low sense of ikigai. Additionally, that a participant’s sense of ikigai will not be predictive of anxiety scores.

## Method

### Participants

An a priori power analysis (*f*^*2*^ = 0.15, α = 0.05, power = 0.80), using G*Power (v3.1), indicated that 92 participants were required for the planned analyses. Inclusion criteria dictated that participants were aged eighteen years or over, self-identified as psychologically fit and healthy, and were either born in or currently live in Europe or North America. Participants were volunteers recruited from a general and student population, who were contacted via [1] the host institution’s psychology Facebook page, [2] Research Participation Scheme (RPS), and [3] social networks known to the researchers via e-mail. Student participants who took part through the RPS were awarded participants points to aid their own participant recruitment. To disseminate the study more widely, interested prospective participants were also asked to forward the study to others who may be interested in taking part.

Thirteen cases were excluded because of incomplete data or not meeting inclusion conditions, e.g. they indicated they were under 18. Overall, data from 94 participants was used (*M*_*age*_ = 41.79 years, *SD* = 15.91 years; RNG_age_ = 18–75 years; 70% female). Fifty-nine participants were employed, 12 were employed yet furloughed (the context of which being the UK government providing employers with 80% of the wages of their employers for them to remain at home during the Coronavirus (COVID-19) pandemic), and 23 were unemployed. Sixty-two participants were non-students and 32 were students.

### Materials

#### Demographics

Participants were asked to indicate their age, sex, employment status, and if they were currently a student.

#### Surveys

The English version of the *Ikigai-9* (Fido et al., 2019) comprises 9 items measuring an individual’s reason for being, exploring emotions about one’s life, attitudes about one’s future, and recognition of one’s existence (e.g. ‘I believe that I have some impact on someone’). Participants indicate how much each statement applies to them on a 5- point.

Likert scale (1 = does not apply to me, 5 = applies to me a lot). Greater scores indicate greater levels of ikigai. The scale has high internal consistency (Fido et al., 2019).

*Beck’s Depression Inventory II (BDI-II)* (Beck et al., 1996) comprises 21 items assessing the presence and intensity of depression symptoms. Every item includes four statements and participants indicate which statement best describes the way they felt over the past week, including today (e.g. I do not feel sad; I feel sad; I am sad all the time and I can’t snap out of it; I am so sad and unhappy that I can't stand it). Items are scored from 0–3. Greater scores indicate increasing depression symptoms. Sound psychometric properties, including internal consistency, have been reported in the general population (Kühner et al., 2007) and student population (Storch et al., 2004).

*The Short Warwick-Edinburgh Mental Well-being Scale* (Stewart-Brown et al., 2009) comprises 7 items that measure psychological functioning and emotional well-being (e.g. ‘I’ve been feeling optimistic about the future’). Participants choose the answer that best describes their experience of the statement over the last two weeks on a 5-point Likert scale (1 = none of the time, 5 = all of the time). Greater scores indicate greater well-being. The scale demonstrates content validity and internal consistency in both general and student populations, lacks the ceiling effect, and is less prone to the social desirability effect seen in comparable scales (Tennant et al., 2007).

*The Generalized Anxiety Disorder 7-item* (GAD-7) scale (Spitzer et al., 2006) comprises 7 items assessing the presence and intensity of anxiety symptoms. Participants choose the option that most accurately illustrates their experience for each statement over the last two weeks (e.g. ‘feeling nervous, anxious, or on edge’). These are the following: not at all (1), several days (2), more than half the days (3), and nearly every day (4). Greater scores indicate greater levels of anxiety. Psychometric evaluation of the GAD-7 indicates it is a reliable and valid measure of anxiety in the student population (Bártolo et al., 2017) and the general population (Löwe et al., 2008).

The BDI-II and GAD-7 were used to overcome the possible limitations of the DASS-21 used within Fido et al. (2019).

### Procedure

It should be made clear that recruitment for the study took place from May 21st – June 9th during the COVID-19 pandemic (2020), and this will be discussed further in the “12” section. Ethical approval was granted by a departmental ethics committee. The survey was completed online through the survey platform Qualtrics^xm^.

On first contact, participants were informed about the study and invited to participate through a URL link. Here, study information was provided and consent, inclusion criteria, and demographic questions were asked. Next, participants completed the *Ikigai-9*, BDI-II, The Short Warwick-Edinburgh Mental Well-being Scale, and GAD-7. Further consent was gained, a debrief provided and participants asked to create a unique code for withdrawal purposes. Participants recruited from both within and outside the host institution were provided with the survey during the same period, limited to 3 weeks, to mitigate potential seasonal biases.

### Analytic Strategy

IMB SPSS 26 and IMB SPSS 27 were used for data analysis. Descriptive statistics and model assumption tests were explored (Field, 2017). Three one-way multivariate analysis of variances (MANOVAs) and follow-up one-way analysis of variances (ANOVAs) were run against sex differences, student status differences, and employment status differences, in age and scores of ikigai, depression, well-being, and anxiety.

Pearson correlations were followed by hierarchical multiple regressions. Participant’s sex, age, employment status, and student status were considered covariates. Three hierarchical models were completed in two stages. Firstly, for consistency and comparability, in accordance with Fido et al. (2019), the variables sex and age were inputted, followed by the new variables of interest employment and student status, ensuring that predictor order was based on previous research (Field, 2017). Secondly, ikigai was added. This indicated how much variation in scores of well-being, depression, and anxiety was explained by ikigai beyond sex, age, employment, and student status.

## Results

### Sex, Student Status and Employment Status Differences in Questionnaire Measures

Sex differences, student status differences, and employment status differences (factors) in age and scores of ikigai, well-being, depression, and anxiety (dependent variables) were explored using one-way MANOVAs. Means and standard deviation values for these tests are reported in Tables 1, 2, and 3. Model assumption tests were run, and data for the variable anxiety, depression, and age were not normally distributed; therefore, a Log10 transformation was performed. There were no significant sex differences in scores of ikigai, well-being, anxiety, depression, or age, *F*(5,88) = 0.889, *p* = 0.492; Wilk’s Λ = 0.952, partial *η2* = . 048. A series of follow-up ANOVAs further confirmed this (for individual significance values, see Table 1). There were significant student status differences in scores of ikigai, well-being, anxiety, depression, or age, *F*(5,88) = 5.353, *p* < 0.001; Wilk’s Λ = 0.767, partial *η2* = . 233. Follow-up ANOVAs indicated that the only students and non-students significantly differed on measures of age, where on average, non-students were older than students (*F* = 23.45, *p* < 0.001) (for individual significance values see Table 2). There were no significant employment status differences in scores of ikigai, well-being, anxiety, depression, or age, *F*(10,174) = 0.1.507, *p* = 0.140; Wilk’s Λ = 0.847, partial *η2* = . 080. However looking at follow-up ANOVAs, there was a significant difference for employment status and age, *F* = 5.257 *p* = 0.007 (for induvial significance values see Table 3). Post hoc tests using the Bonferroni correction revealed that the mean age for individuals employed furloughed was significantly less than the mean age of unemployed individuals and employed individuals (*p* = 0.005 and *p* = 0.045).

**Table 1:** Descriptive statistics for scores with between sex comparisons

	Total *M* (*SD*)	Males (*n* = 28) *M* (*SD*)	Females (*n* = 66) *M* (*SD*)	*p*
Age	1.58(0.17)	1.57 (0.18)	1.60 (0.17)	0.43
Ikigai	38.55 (4.82)	37.57 (5.09)	38.97 (4.68)	0.20
Depression	0.92 (0.33)	0.90 (0.32)	0.92 (0.34)	0.78
Well-being	23.15 (4.31)	23.18 (4.11)	23.14 (4.42)	0.97
Anxiety	1.11 (0.15)	1.09 (0.15)	1.12 (0.15)	0.37

^Significant differences highlighted in bold: no significant difference was detected^.

**Table 2:** Descriptive statistics for scores with between student status comparisons

	Total *M* (*SD*)	Non-Student (*n* = 62) *M* (*SD*)	Student (*n* = 32) *M* (*SD*)	*p*
Age	1.59 (0.17)	1.64 (0.15)	1.48 (0.16)	** < 0.001**
Ikigai	38.55 (4.82)	38.27 (4.77)	39.09 (4.95)	0.44
Depression	0.92 (0.33)	0.92 (0.28)	0.91 (0.42)	0.91
Well-being	23.15 (4.31)	23.65 (3.56)	22.19 (5.42)	0.12
Anxiety	1.11 (0.15)	1.10 (0.15)	1.12 (0.15)	0.44

^Significant differences highlighted in bold^.

**Table 3:** Descriptive statistics for scores with between employment status categories

	Total *M* (*SD*)	Employed (*n* = 59)* M* (*SD*)	Employed Furloughed (*n* = 12)* M* (*SD*)	Unemployed (*n* = 23)* M* (*SD*)	*p*
Age	1.59 (0.17)	1.60 (0.14)	1.46 (0.18)	1.65 (0.20)	**0.007**
Ikigai	38.55 (4.82)	38.80 (4.80)	37.08 (5.93)	38.70 (4.32)	0.53
Well-being	2 23.15 (4.31)	23.73 (4.02)	21.25 (5.03)	22.65 (4.49)	0.16
Depression	0.92 (0.33)	0.89 (0.36)	0.97 (0.28)	0.97 (0.28)	0.56
Anxiety	1.11 (0.15)	1.10 (0.15)	1.15 (0.14)	1.10 (0.14)	0.52

^Significant differences highlighted in bold^.

### Predicting Well-Being, Depression, and Anxiety

Model assumptions for multiple regression analysis were met (see https://osf.io/4pbz9/?view_only=8be00896fd7a439a96dc0d1738d06e12). Ikigai significantly negatively correlated with measures of depression and anxiety, and significantly positively correlated with measures of well-being. Scores on depression and anxiety were positively correlated with one another and negatively correlated with well-being (Table 4). Three hierarchical multiple regression analyses were conducted. For each analysis, age, sex (0 = male, 1 = female), employment status (employed vs furloughed, employed vs unemployed), and student status (0 = student, 1 = non-student) were entered during step 1, and ikigai was entered during step 2. The dependant variables were scores on well-being, depression, and anxiety.

**Table 4:** Correlations between ikigai, well-being, depression, and anxiety

	Well-being	Depression	Anxiety
*Correlations*			
Ikigai	58***	− 0.45***	− 0.22**
Well-being	-	− 0.67***	− 0.46***
Depression		-	0.66***
Anxiety			-

^*p < .05; **p < .01; ***p < .001^

In step 1, neither age, sex, employment status, nor student status significantly contributed to the prediction of well-being, depression, or anxiety (Table 5). When introducing scores on ikigai in step 2, this significantly (*p* < 0.001) explained an extra 33% variance in well-being (positive association) and an extra 21% variance in depression (negative association). While ikigai explained an addition 0.5% in variation in anxiety, this was not a significant increase (*p* = 0.149; Table 5).

**Table 5:** Standardised regression coefficients and 95% confidence intervals between. Ikigai, well-being, depression, and anxiety

		Well-being		Depression		Anxiety	
*Standardised regression coefficients and 95% confidence intervals*							
Step 1	Sex	0.04	[− 1.56, 2.26]	− 0.07	[− 4.04, 2.02]	− 0.11	[− 3.24, 0.95]
	Age	0.24	[0.00, 0.13]	− 0.12	[− 0.15, 0.05]	− 0.18	[− 0.12, 0.02]
	Employed Furloughed	− 0.14	[− 4.60, 0.00]	0.09	[− 2.55, 6.26]	0.09	[− 1.81, 4.29]
	Unemployed	− 0.16	[-3.74, 0.51]	0.07	[− 2.33, 6.26]	− 0.02	[− 2.15,2.53]
	Student status	0.03	[− 1.80, 2.33]	− 0.04	[− 3.83, 2.72]	0.01	[− 2.12, 2.40]
	Model	*F*(5,88) = 1.878, *p* = 0.106	*F*(5,88) = 0.654, *p* = 0.659	*F*(5,88) = 0.942, *p* = 0.458			
	*R*^2^	0.10		0.04		0.05	
Step 2	Sex	0.10	[− 0.58, 2.50]	− 0.12	[-4.47, 0.96]	− 0.14	[− 3.46, 0.67]
	Age	0.19	[0.00, 0.10]	− 0.08	[− 0.12, − 0.06]	− 0.16	[− 0.11, 0.02]
	Employed Furloughed	− 0.07	[− 3.08, 1.40]	0.03	[− 3.30,4.60]	0.06	[− 2.18, 3.84]
	Unemployed	− 0.15	[− 3.17, 0.24]	0.06	[− 2.12, 3.88]	0.01	[− 2.16, 2.04]
	Student Status	0.12	[− 0.62, 2.71]	− 0.11	[− 4.44, 1.44]	− 0.02	[− 2.435, 2.041]
	Ikigai	0.59***	[− 0.62, 2.71]	− 0.47***	[− 0.90, − 0.38]	− 0.23*	[− 0.42, − 0.02]
	Model	*F*(6,87) = 10.862, *p* < 0.001	*F*(6,87) = 4.70, *p* < 0.001	*F*(6,87) = 1.628, *p* = 0.149			
	*R*^2^	0.43		0.25		0.10	

^*p < .05; **p < .01; ***p < .001^

## Discussion

Having a sense of ikigai has been linked to physical and psychological well-being in Japanese samples (Mori et al., 2017; Okamoto & Harasawa, 2009; Sone et al., 2008; Tanno et al., 2009). However, to date, only one study has investigated ikigai in a Western sample (Fido et al., 2019). The present study looked to replicate and extend the aforementioned literature and to further determine if ikigai can predict measures of well-being, depression, and anxiety in a Western non-clinical adult sample. Moreover, it looked to identify differences in levels of ikigai among sex, student status, and employment status. Ikigai was found to be predictive of well-being and depression, but not anxiety. Additionally, no differences were found in levels of ikigai for sex, student status, and employment status.

Data reported here indicates that ikigai predicts self-reported levels of well-being, depression, and anxiety. After accounting for the demographic variables age, sex, employment, and student status, ikigai significantly (and positively) predicted 31% of the variance seen in well-being and significantly (and negatively) predicted 21% of the variance seen in depression. Therefore, and confirming our hypotheses, the greater the presence of ikigai reported, the greater the well-being and the lower the depressive symptoms. Additionally, ikigai negatively predicted 0.5% of variance in anxiety; however, this result was not statistically significant, meaning that in this sample, after accounting for covariates, greater ikigai did not lead to reduced anxiety.

In line with Fido et al. (2019), this study found that ikigai was predictive of well-being and depression. However, in comparison to Fido et al. (2019), ikigai in our study explained 16% more variance in well-being and a further 19% of variance in depression. While this study used a different measure of depression, namely *Beck’s Depression Inventory II* (Beck et al., 1996), from Fido et al. (2019), it used the same measure of well-being, namely *The Short Warwick-Edinburgh Mental Well-being Scale* (Stewart-Brown et al., 2009). It is useful to see that the findings of Fido et al. (2019) are replicated regardless of the measure used, and it could be argued that the larger proportion of variance presently explained is a direct result of using a more comprehensive measure—here *Beck’s Depression Inventory II* (Beck et al., 1996). The additional variance could also be explained by trends of ikigai being higher than in previous samples. In contrast to the average ikigai score in a Japanese sample (33.3 ± 5.3) (Imai et al., 2012) and a UK sample (32.87 ± 7.91) (Fido et al., 2019), the present study found ikigai was noticeably higher (38.55 ± 4.82). These findings could be due to the study coinciding with the (COVID-19) pandemic, a period during which it is possible that people were more actively engaged in a re-evaluation of what makes life worth living, and thereby influencing their sense of ikigai (Hughes, 2020). However, more research would be needed to test the accuracy of this hypothesis. Further, unlike the study by Fido et al. (2019) which concentrated exclusively on the UK population, this study sampled from both Europe and North America. The present finding of increased ikigai trends could indicate that diversity in ikigai exists across the West. However, as location details of the participants were not presently collected, further qualitative and quantitative research is needed to explore this, in which participant location is explicitly captured.

Surprisingly, neither age, sex, employment status, nor student status significantly predicted well-being, anxiety, or depression. This contrasted with previous studies which found that these factors were predictive of well-being, anxiety, and depression (Boyd et al., 2015; Gray et al., 2019; Ibrahim et al., 2013; Jefferis et al., 2011; McManus et al., 2016; Neves & Hillman, 2017; Paul & Moser, 2009; Steptoe et al., 2015). These findings may be due to the COVID-19 pandemic; however, research exploring the impact of COVID-19 indicated differences still exist in mental well-being, depression, and anxiety across age, gender, and employment (Jia et al., 2020; Kazmi et al., 2020; Smith et al., 2020). COVID-19 therefore may not be the underlining explanation. Alternatively, as the sample size is smaller than equivalent research, it may not have sufficient power to detect effects, should they exist (Field, 2017).

It is noteworthy that before demographic variables were accounted for, ikigai did significantly and negatively predict anxiety. However, following the addition of the covariates, this relationship did not hold. This confirms our hypothesis that no association between ikigai and anxiety would be found and is consistent with findings by Fido et al. (2019). Further it highlights that even after using a different measure of anxiety, namely *The Generalized Anxiety Disorder 7-item* (GAD-7) (Spitzer et al., 2006) than Fido et al. (2019), a relationship between ikigai and anxiety still does not exist. Interestingly, research by Ishida and Okada (2006) on purpose in life did find a relationship between ikigai and anxiety. However, Ishida and Okada (2006) focused on trait anxiety, while the present study explored state anxiety. This suggests that while ikigai may potentially be predictive of anxious personalities (i.e. trait anxiety), it may not be predictive of temporary changes in anxiety (i.e. state anxiety). Alternatively, this difference may be explained by cultural characteristics. Ishida and Okada’s study (2006) was conducted with a Japanese sample, who are rather sensitive to anxiety (Hofstede et al., 2010). Therefore, having a strong sense of ikigai was directly associated with anxiety, which many Japanese were highly aware of, in their study.

Consistent with Fido et al. (2019), no significant differences were found in ikigai measures for sex. There were also no significant differences in ikigai based on employment or student status. This indicates that outward status, be it employment or education, does not necessarily offer individuals the ‘purpose in life’ consistent with ikigai. This finding highlights that ikigai represents an individual standard of personal happiness as opposed to one externally set (Shirai et al., 2006).

There were some limitations of the present study. Firstly, the Ikigai-9 scale used in this study has not been assessed for stability (Fido et al., 2019). It is important that test–retest validation is undertaken to better understand any protective role it may have in mental health. Secondly, causation cannot be inferred as the study is cross-sectional. Finally, with only preliminary research into COVID-19, it is difficult to fully appreciate how this may have affected the results. For example, Harper et al. (2020) found that general anxiety is increasing during COVID-19, and therefore, the levels of anxiety reported here may differ if the study was repeated at a different time.

This is the second time the English-translated Ikigai-9 has been used. As this field of research develops, focus could be given to specific subtypes of anxiety, e.g. social phobia, directing research to the treatment of specific disorders. Additionally, more work is needed looking at how a sense of ikigai leads to health benefits. Ikigai may be associated with reduced smoking and drinking (Tanno et al., 2009), outcomes which are themselves associated with positive well-being and lower depression and anxiety (Awaworyi Churchill & Farrell, 2017; Barros et al., 2015; Moylan et al., 2013). Further mediating factors such as exercise could also be explored. While further research is needed to understand how ikigai is developed, it has been suggested ikigai feelings can be gained in both childhood (Ishida, 2012) and adulthood (Kono and Walker, 2019). Further research could also look to explore the precise mechanism linking ikigai and psychological well-being and depression, which is still unknown, and look to develop a theoretical framework to address this. These findings alongside those presented here support the importance for investigating ikigai in the West and the need for further exploration of ikigai as a potential means for improving psychological well-being. Developing interventions that help people foster an awareness of and enhance their ikigai could improve mental health. Improvements to mental health benefit the individual and society from a biopsychosocial and finical perspective. Globally the financial cost of poor mental health is predicted to be 2.5 trillion dollars per year (The Lancet Global Health, 2020), and therefore, identifying cost-effective interventions that may improve mental health is warranted.

Overall, this study has found that ikigai is predictive of well-being, and depression in a Western adult population. This indicates that there is a conceptual gap in the Western understanding of mental health and that much can be, and should be, learned from Eastern cultures when exploring ‘purpose in life’.
